# Gain in Brain Immunity in the Oldest-Old Differentiates Cognitively Normal from Demented Individuals

**DOI:** 10.1371/journal.pone.0007642

**Published:** 2009-10-29

**Authors:** Pavel Katsel, Weilun Tan, Vahram Haroutunian

**Affiliations:** 1 Department of Psychiatry, Mount Sinai School of Medicine, New York, New York, United States of America; 2 Department of Psychiatry, James J. Peters VA Medical Center, Bronx, New York, United States of America; Massachusetts General Hospital and Harvard Medical School, United States of America

## Abstract

**Background:**

Recent findings suggest that Alzheimer's disease (AD) neuropathological features (neuritic plaques and NFTs) are not strongly associated with dementia in extreme old (over 90 years of age) and compel a search for neurobiological indices of dementia in this rapidly growing segment of the elderly population. We sought to characterize transcriptional and protein profiles of dementia in the oldest-old.

**Methods and Findings:**

Gene and protein expression changes relative to non-demented age-matched controls were assessed by two microarray platforms, qPCR and Western blot in different regions of the brains of oldest-old and younger old persons who died at mild or severe stages of dementia. Our results indicate that: i) consistent with recent neuropathological findings, gene expression changes associated with cognitive impairment in oldest-old persons are distinct from those in cognitively impaired youngest-old persons; ii) transcripts affected in young-old subjects with dementia participate in biological pathways related to synaptic function and neurotransmission while transcripts affected in oldest-old subjects with dementia are associated with immune/inflammatory function; iii) upregulation of immune response genes in cognitively intact oldest-old subjects and their subsequent downregulation in dementia suggests a potential protective role of the brain immune-associated system against dementia in the oldest-old; iv) consistent with gene expression profiles, protein expression of several selected genes associated with the inflammatory/immune system in inferior temporal cortex is significantly increased in cognitively intact oldest-old persons relative to cognitively intact young-old persons, but impaired in cognitively compromised oldest-old persons relative to cognitively intact oldest-old controls.

**Conclusions:**

These results suggest that disruption of the robust immune homeostasis that is characteristic of oldest-old individuals who avoided dementia may be directly associated with dementia in the oldest-old and contrast with the synaptic and neurotransmitter system failures that typify dementia in younger old persons.

## Introduction

The continued expansion of the elderly population resulting from recent advances in medical treatment, improved nutrition and reduced birth rate accompanied by elevated age-related risk of brain disorders such as dementia and Alzheimer's disease (AD) have prompted considerable interest in the study of the aging human brain. These advances in life expectancy have not only led to an increase in the proportion of the population that is aged, but also in the numbers of persons who can be classified as the oldest-old, variably defined as persons over the age of 85 or 90. US Census Bureau data and projections [1], [2] show that the number of American over the age of 85 (4.4 million in 2001) will rise significantly by 2010 to 5.8 million and will quadruple to 19.3 million by 2050.

The high frequency of dementia incidence in the elderly with reported doubling every five years from ages 65 to 85 [3] would predict that some aspect of dementia will afflict most individuals by age of 100 [4], [5]. However, clinical and neuropathological studies suggested a decline or plateau in the incidence of dementia in the oldest-old [6]–[11]. Moreover, epidemiological studies indicate that the rate of increase in dementia prevalence falls in octogenarians and levels off at about 40% at age 95 [9] suggesting a selective survival effect [12]. These data and socio-demographic studies of nonagenarians and centenarians (rev. by Imhof et al [13]) support the possibility that oldest-old persons may represent a select group with a decreased rate of aging and increased resistance to biological and physiological stresses and age-related diseases, such as cancer, stroke and cardiovascular disease. However, even if the rate of dementia stabilizes beyond age 85, the numbers of oldest-old persons with dementia are likely to rise to 8–10 million by 2050.

Neuropathological studies laid the foundation for our understanding of the development of the dementing processes in AD and helped define the relationship between neuropathological lesion severity and the magnitude of cognitive impairment. Neuritic plaques (NPs) and neurofibrillary tangles (NFTs), as well as synaptic loss in the temporal neocortex, the entorhinal cortex and the hippocampus were found to be associated with the severity of cognitive deficits in younger AD patients [14]–[16]. In contrast to younger cases, persons in their 60s–80s, the relatively limited number of studies in nonagenarians and centenarians have found considerable differences in the magnitude and regional/sub-regional distribution of NPs and NFTs in cognitively impaired cases [17]–[20] (but see [21] for contrasting findings). Additionally, there was no association between AD neuropathology and neuronal loss in CA1-3 areas of hippocampus (HIPP) and entorhinal cortex in oldest-old AD cases [22]–[24]. Moreover, quantitative studies have found that the neuropathological correlates of cognitive impairment in AD appear to be more variable and often significantly less profound in the oldest-old than in younger persons [17]–[19], [22]–[29]. The observed reduced NP and NFT densities in oldest-old individuals diagnosed clinically with AD raises the question of whether different underlying neurobiological mechanisms are associated with cognitive impairment in oldest-old persons as compared to younger old persons with dementia.

Significant progress has been made in understanding the molecular biological abnormalities in the brains of persons with AD [30]–[33]. Despite the substantial interest in transcriptional abnormalities in AD, most studies have approached abnormal gene expression in AD by examining gene expression profiles in groups selected on the basis of specific neuropathology or cognitively distinct stages of the disease rather than stratifying demented individuals according to their age. A few studies have assessed gene expression in the brain throughout the lifespan (13–106 y.o.) and have shown that significant changes in co-regulated sets of genes implicated in aging occur early in adult life and continue progressively during aging [34]–[36]. The expression of genes that play a role in synaptic plasticity, vesicular/protein transport, neurotransmitter release are generally reduced in the aged cerebral cortex, whereas the expression of genes associated with the stress responses, DNA damage and repair and immune/inflammation are frequently increased [34]. A recent study of cognitively intact non-AD persons that compared gene expression profiles in the three most vulnerable regions of the AD brain: HIPP, entorhinal and superior temporal cortex as well as less vulnerable – postcentral gyrus - across the lifespan (22–99 y.o.) also showed increased immune activation around the sixth to seventh decades [35]. The expression of similar categories of genes related to synaptic function, DNA damage and inflammation with similar directional changes were also identified in several cortical areas and the hippocampus of elderly persons diagnosed with AD or mild cognitive impairment [37]–[40].

The growing body of evidence showing dissociation between the neuropathological indices of AD from dementia in the extreme elderly prompted the current study of the molecular biological substrates of dementia in this rapidly growing segment of the elderly population. The presence, absence and severity of dementia was assessed by the clinical dementia rating (CDR) rating scale [41]. Gene and protein expression profiles of multiple brain regions from autopsied cognitively normal (CDR0), mildly cognitively impaired (CDRs 0.5–1) and severe dementia persons (CDRs 4–5) with ages ranging between 60 and 86 years, were compared to the profiles of similar groups of oldest-old individuals ranging from 87 to 107 years of age. These cases included persons who evidenced either no significant neuropathology or only NP and NFT neuropathology. Cases with clinical conditions known to be associated with cognitive impairment other than AD (e.g., schizophrenia, major depression) and cases with neuropathologies associated with conditions other than AD (e.g. Lewy bodies with or without NPs and NFTs), or significant cerebrovascular lesions, were excluded.

## Results

### Median age split groups of cognitively impaired patients showed different sets of affected genes

**Figure 1:**
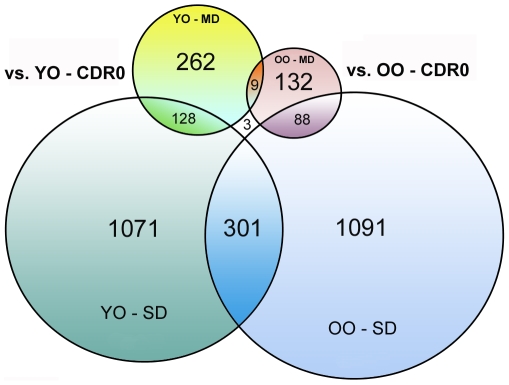
Numbers of probe sets of differentially expressed genes in cognitively impaired groups of two median split age cohorts compared to corresponding age matching controls. YO- young-old (≤86 y.o.); OO –oldest-old (≥87 y.o.); MD (CDRs 0.5–1) and SD (CDRs 4–5). Overlapping regions include common number of probe sets. Number in the center outside of all terms indicates probe sets common across all of the tested groups.

All samples in the microarray dataset were divided in two major age categories based on the median age (87 y.o.) of all of the subjects: youngest-old (YO; ≤86 y.o.) and oldest-old (OO; ≥87y.o.) in order to analyze gene expression changes in clinically and neuropathologically distinct age groups of cognitively impaired subjects and controls (Table 1). In order to identify genes affected in cognitively impaired individuals and to test whether similar genes or pathways are affected during the course of dementia in each age group CDR classified cognitively impaired subjects were sorted into two additional groups: mild dementia (MD, CDRs 0.5–1) and severe dementia (SD, CDRs 4–5). Each of these groups was then compared to age- matched cognitively normal controls within their respective age categories. Filtering conditions were: fold change >1.3 and p<0.05; present call >70%. As shown in Fig. 1, samples from YO and OO MD groups had significantly smaller number (4 to 8 times) of differentially expressed probe sets as compared to SD groups (probe sets are defined as probes on the microarray that identify a single mRNA transcript). The number of common probe sets between MD groups in the two age categories, however, were strikingly small (below 7%) as compared to the overlap between mildly and severely demented groups within each age category: 48% for YO and 66% for OO individuals, indicating very little similarity between mildly cognitively impaired persons in the two age groups. As dementia progressed to terminal stages, this overlap between the two age groups remained low: approximately 28%. Removing subjects with questionable dementia (CDR0.5) from the MD groups did not significantly affect the overall total number of affected genes in each age group nor the overlapping probe sets, and even strengthened the percent-wise separation between the two age cohorts (data not shown).

**Table 1:** Sample characteristics.

**Dementia Groups/arrays**	**CNT 86−**	**MD 86−**	**SD 86−**	**CNT 87+**	**MD 87+**	**SD 87+**
Subjects #	15	6	13	7	15	15
Total samples/arrays	144	52	106	72	143	69
Sex (M/F)	7/8	2/4	4/9	1/6	3/12	3/12
Age (years)	75.2±8.6	76.8±8.4	80.8±7.1	94.2±4.6	90.8±3.9	92.9±4.8
Brain pH	6.55±0.25	6.34±0.09	6.41±0.28	6.49±0.26	6.35±0.28	6.34±0.22
PMI (h)	8.68±6.18	4±1.2	5.31±5.26	4.03±1.53	5.45±5.28	3.55±1.40
**Dementia Groups/qPCR**	**CNT 86−**	**MD 86−**	**SD 86−**	**CNT 87+**	**MD 87+**	**SD 87+**
Total subjects (N)	7	7	5	6	13	17
Sex (M/F)	2/5	3/4	3/2	3/3	3/10	1/16
Age (years)	77.3±5.7	77.4±6.5	79.2±9.7	89.8±2.9	90.5±3.5	92.9±4.4
Brain pH	6.29±0.32	6.34±0.25	6.26±0.45	6.27±0.37	6.33±0.24	6.35±0.30
PMI (h)	8. 7±6.2	3.5±1.2	8.3±8.8	4.1±0.1	4.2±1.9	4.7±3
**Dementia Groups/WB**	**CNT 86−**	**MD 86−**	**SD 86−**	**CNT 87+**	**MD 87+**	**SD 87+**
Total subjects (N)	10	-	9	10	-	10
Sex (M/F)	4/6	-	3/6	3/7	-	3/7
Age (years)	79.7±6.7	-	79.0±7.6	93.2±4.8	-	93.8±4.7
Brain pH	6.43±0.41	-	6.38±0.14	6.5±0.24	-	6.35±0.30
PMI (h)	7.3±4.6	-	5.2±4.9	4.2±1.5	-	4.4±3.6

† Microarrays were performed in 15 brain regions (BAs: 8, 10, 44, 46, 4, 32/24, 23/31, 20, 21, 22, 36/28, 38, 7, 17 and HIPP). qPCR and western blotting (WB) studies were performed in the inferior temporal cortex –BA20. 86− (Youngest-old (60–86 y.o.)); 87+ (Oldest-old (87–103 y.o.)); MD- mild dementia (CDR 0.5–1); SD-severe dementia (CDR 4–5).

### Pathway analysis showed distinct molecular profiles in two cognitively impaired cohorts stratified by age

The Ingenuity Pathway Analysis algorithm was used to determine which signaling pathways were affected in the groups of cognitively impaired subjects in each of the two age cohorts (Tables 2 and 3). As expected from the previous analysis (Venn Fig. 1) that indicated very little overlap between the two age cohorts of cognitively impaired groups of subjects, there were only three common pathways observed. The granulocyte/macrophage colony-stimulating factor (GM-CSF) signaling pathway (related to innate immune response) appeared among the top scored pathways in individuals with mild dementia of both age groups. Oxidative phosphorylation and synaptic LTP were the other two common pathways significantly affected in individuals with severe dementia in the two age categories. Additionally, there was a high level of consistency within the youngest-old cognitively impaired groups of subjects. Overall, dementia-associated genes with altered expression in the young-old (Table 2) were predominantly represented by the signaling, but not by the metabolic, pathways typically associated with synaptic function, such as GABA and glutamate receptor signaling, synaptic long term potentiation (LTP), and pathways associated with neural development, including neuregulin, IGF1, VEGF, PDGF, PI3K/Akt, axonal guidance and calcium signaling. Other identified pathways were previously implicated in brain aging or neurodegenerative disorders and included IGF1, NF-kB, chemokine, cell cycle, nitric oxide, NRF2-mediated oxidative stress response and glucocorticoid receptor signaling and pathways related to neurodegeneration – hypoxia, Huntington's, Parkinson's diseases and amyotrophic lateral sclerosis signaling. Generally, broad diversity of listed signaling pathways in subjects with mild dementia was narrowed, while percentage of the affected genes relative to the total number of genes in these pathways was increased in severe cognitively impaired young-old subjects.

**Table 2:** Canonical pathways affected in cognitively impaired youngest-old (≤86 y.o.).

*Pathway Name*	*-Log(P-value) MD*	*Ratio MD*	*-Log(P-value) SD*	*Ratio SD*
**GABA Receptor Signaling**	**5.09**	**1.32E-01**	**7.00**	**2.64 E-01**
**Glutamate Receptor Signaling**	**3.44**	**8.96E-02**	**2.31**	**1.34E-01**
**IGF-1 Signaling**	**3.21**	**7.61E-02**	**1.56**	**1.09E-01**
**Nitric Oxide Signaling in the Cardiovascular System**	**2.98**	**7.06E-02**	**2.21**	**1.18E-01**
**Synaptic Long Term Potentiation**	**2.73**	**6.31E-02**	**3.52**	**1.44E-01**
**Huntington's Disease Signaling**	**2.52**	**4.31E-02**	**4.47**	**1.21E-01**
Neuregulin Signaling	2.50	6.59E-02	*ns*	*ns*
Cell Cycle: G2/M DNA Damage Checkpoint Regulation	2.35	9.30E-02	*ns*	*ns*
NF-kB Signaling	1.99	4.90E-02	*ns*	*ns*
VEGF Signaling	1.88	5.43E-02	*ns*	*ns*
**Amyotrophic Lateral Sclerosis Signaling**	**1.84**	**4.85E-02**	**1.64**	**1.07E-01**
Glutamate Metabolism	1.83	3.85E-02	*ns*	*ns*
PPAR Signaling	1.82	5.26E-02	*ns*	*ns*
Glucocorticoid Receptor Signaling	1.78	3.40E-02	*ns*	*ns*
PI3K/AKT Signaling	1.74	3.98E-02	*ns*	*ns*
GM-CSF Signaling	1.72	6.45E-02	*ns*	*ns*
Xenobiotic Metabolism Signaling	1.68	3.60E-02	*ns*	*ns*
Hypoxia Signaling in the Cardiovascular System	1.63	5.63E-02	*ns*	*ns*
Synaptic Long Term Depression	1.61	3.68E-02	*ns*	*ns*
**Parkinson's Signaling**	**1.57**	**1.18E-01**	**1.99**	**2.35E-01**
NRF2-mediated Oxidative Stress Response	1.54	3.89E-02	*ns*	*ns*
**ERK/MAPK Signaling**	**1.52**	**3.54E-02**	**1.33**	**8.41E-02**
PDGF Signaling	1.52	5.41E-02	*ns*	*ns*
**Cardiac β-adrenergic Signaling**	**1.49**	**3.85E-02**	**1.39**	**8.46E-02**
**G-Protein Coupled Receptor Signaling**	**1.39**	**3.52E-02**	**1.59**	**9.05E-02**
Oxidative Phosphorylation	*ns*	*ns*	4.28	1.20E-01
Calcium Signaling	*ns*	*ns*	3.53	1.08E-01
cAMP-mediated Signaling	*ns*	*ns*	2.57	1.13E-01
Axonal Guidance Signaling	*ns*	*ns*	2.50	8.53E-02
Chemokine Signaling	*ns*	*ns*	2.04	1.33E-01
Actin Cytoskeleton Signaling	*ns*	*ns*	1.85	8.61E-02
Mitochondrial Dysfunction	*ns*	*ns*	1.45	7.88E-02
Leukocyte Extravasation Signaling	*ns*	*ns*	1.40	8.51E-02

† Fischer's exact test was used to calculate p-values; the ratios are between the numbers of genes from the dataset that mapped to the pathway to the total numbers of genes in the canonical pathway. MD  =  CDRs 0.5–1; SD  =  CDRs 4–5; ns- Non significant. Pathways are sorted in descending order based on significance for MD group. Common pathways between the two young-old and oldest-old dementia groups are in bold font.

**Table 3:** Canonical pathways affected in cognitively impaired oldest-old (≥87 y.o.).

*Pathway Name*	*-Log(P-value) MD*	*Ratio MD*	*-Log(P-value) SD*	*Ratio SD*
**Antigen Presentation Pathway**	**7.11**	**3.33E-01**	**1.40**	**2.82E-01**
Complement System	3.63	1.67E-01	*ns*	*ns*
Natural Killer Cell Signaling	2.31	4.55E-02	*ns*	*ns*
Protein Ubiquitination Pathway	1.98	4.93E-02	*ns*	*ns*
Acute Phase Response Signaling	1.69	4.07E-02	*ns*	*ns*
Interferon Signaling	1.68	1.38E-01	*ns*	*ns*
GM-CSF Signaling	1.61	8.06E-02	*ns*	*ns*
Fc Epsilon RI Signaling	1.45	4.00E-02	*ns*	*ns*
Methane Metabolism	1.40	3.08E-02	*ns*	*ns*
IL-4 Signaling	1.36	5.88E-02	*ns*	*ns*
Oxidative Phosphorylation	*ns*	*ns*	2.39	2.15E-01
Synaptic Long Term Potentiation	*ns*	*ns*	1.32	1.62E-01

† Fischer's exact test was used to calculate p-values; the ratios are between the numbers of genes from the dataset that mapped to the pathway to the total numbers of genes in the canonical pathway. ns- Non significant. Common pathways between the two young-old and oldest-old dementia groups are in bold font.

Clear distinctions were observed in the oldest-old cohort with mild dementia. Changes in the expression of signaling pathways related to neural function as seen in the young-old subjects (Table 3) were absent. The majority of the pathways in this group were related to the innate immune response pathways and immune function, which were predominantly downregulated. Only three canonical pathways remained in the cognitively impaired oldest-old subjects. These pathways were; oxidative phosphorylation, antigen presentation and synaptic LTP.

### Cognitively intact oldest-old subjects showed increased expression of immune response genes relative to cognitively intact youngest-old

**Figure 2:**
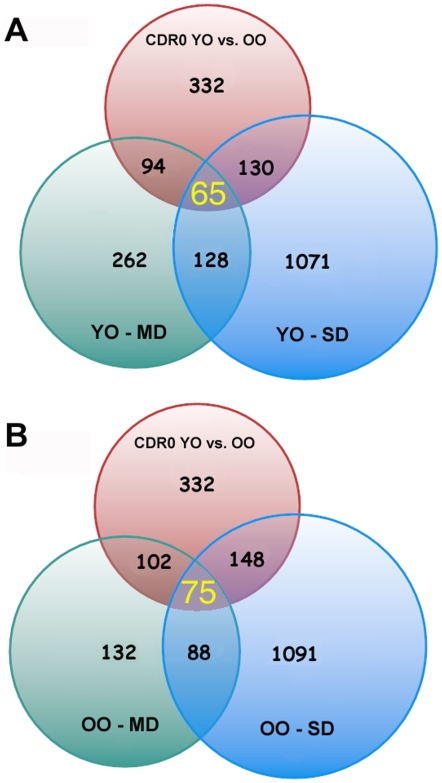
Numbers of common probe sets in differentially expressed genes between two age distinct cognitively intact subjects (CDR0) compared to cognitively impaired groups for each age cohorts. **A.** Venn diagram represents young-old cohort (YO; ≤86 y.o.). **B.** Venn diagram represents oldest-old cohort (OO; ≥87 y.o.).

Because of the clear distinction in genes and pathways associated with cognitive impairment in young-old and oldest-old individuals, we sought to determine whether the gene expression profiles of cognitively intact oldest-old persons were also different from those of young-old cognitively intact persons. We compared cognitively intact young-old individuals to cognitively intact oldest-old persons. Under the same filtering conditions as for the cognitively impaired groups 332 probe sets showed significantly altered expression in oldest-old cognitively intact subjects relative to cognitively intact young-old persons. As shown in Fig. 2, 65 of these 332 “age-associated” probe sets were also differentially expressed in cognitively impaired young-old persons and 75 of the 332 probe sets were differentially expressed in cognitively impaired oldest-old persons. Strikingly, every single transcript among these common genes, i.e. genes whose expression was changed as a function of aging (cognitively intact young-old vs. cognitively intact oldest-old) and as a function of dementia (cognitively intact vs., cognitively impaired) was changed in the opposite direction in the cognitively impaired oldest-old subjects vs. young-old persons. Interferon-induced protein with tetratricopeptide repeats 3 (IFIT3) was the only exception to the above. Gene ontology classification of these common probe sets from two age categories (Table 4), additionally, confirmed distinctive sets of genes involved in different biological processes in the youngest-old versus oldest-old groups.

**Table 4:** GO biological processes categories of common genes (Fig. 2) in youngest-old (≤86 y.o.) and in oldest-old (≥87 y.o.) persons.

***GO ID***	***GO NAME/≤86y.o. (65 genes)***
GO:0006811	ion transport
GO:0007268	synaptic transmission
GO:0019226	transmission of nerve impulse
GO:0007267	cell-cell signaling
GO:0050877	neurological system process
GO:0007186	G-protein coupled receptor protein signaling pathway
GO:0016192	vesicle-mediated transport
GO:0007010	cytoskeleton organization and biogenesis
GO:0042221	response to chemical stimulus
***GO ID***	***GO NAME/≥87y.o. (75 genes)***
GO:0016064	immunoglobulin mediated immune response
GO:0006959	humoral immune response
GO:0002252	immune effector process
GO:0042221	response to chemical stimulus
GO:0019883	antigen processing and presentation of endogenous antigen
GO:0006954	inflammatory response
GO:0019724	B cell mediated immunity
GO:0002541	activation of plasma proteins during acute inflammatory response
GO:0051239	regulation of multicellular organismal process

The same canonical immune function pathways that were identified in the oldest-old individuals with mild dementia (Table 3) were affected in the oldest-old cognitively intact persons (Table 5), but in opposite directions (see above). Additional pathways identified were two metabolic pathways: tyrosine and pyrimidine metabolism and glucocorticoid signaling, pathway frequently implicated in brain aging.

**Table 5:** Canonical pathways affected in cognitively intact oldest-old (≥87 y.o.) compared to cognitively intact youngest-old (≤86 y.o.).

*Pathway Name*	*-Log(P-value)*	*Ratio*
**Antigen Presentation Pathway**	**6.76**	**4.36E-01**
**IL-4 Signaling**	**3.05**	**1.47E-01**
**Natural Killer Cell Signaling**	**3.05**	**1.00E-01**
**Complement System**	**2.33**	**1.11E-01**
**Acute Phase Response Signaling**	**2.21**	**7.56E-02**
Tyrosine Metabolism	1.90	3.24E-02
**Fc Epsilon RI Signaling**	**1.72**	**9.00E-02**
Pyrimidine Metabolism	1.68	3.10E-02
Glucocorticoid Receptor Signaling	1.39	6.04E-02

† Fischer's exact test was used to calculate p-values; the ratios are between the numbers of genes from the dataset that mapped to the pathway to the total numbers of genes in the canonical pathway. Immune-related pathways are in bold font.

The majority of the 75 probe sets (Fig. 2) that were differentially expressed in the brain of cognitively intact and cognitively impaired oldest-old, relative to cognitively intact young-old persons, were related to immune response pathways (Table 5). Table 6 lists 37 genes after removal of redundant probes and genes of unknown function from the 75 probe sets differentially expressed in the brain of cognitively intact oldest-old individuals. These genes showed strong upregulation in cognitively intact oldest-old persons relative to young-old controls (Table 6 and Fig. S1). The vast majority of these genes were profoundly downregulated in cognitively impaired oldest-old persons (Table 6 and Fig. S1).

**Table 6:** Significantly affected genes (*p*<0.05) in the oldest-old (≥87 y.o.) cohort in comparison to cognitively intact youngest-old (≤86 y.o.) subjects.

*Gene Name*	*Symbol*	*CDR 0*	*MD (CDR0.5–1)*	*SD (CDR 4–5)*
**Major histocompatibility complex, class II, DP alpha 1**	**HLA-DPA1**	10.35	−7.03	−5.67
**TYRO protein tyrosine kinase binding protein (DAP12)**	**TYROBP**	9.83	−5.83	−4.38
**Complement component 3**	**C3**	9.81	−5.31	−4.39
**Major histocompatibility complex, class II, DR beta 1**	**HLA-DRB1, 3**	8.59	−5.48	−3.97
**Glycoprotein (transmembrane) nmb**	**GPNMB**	8.57	−6.96	−4.89
**Complement component 1, q subcomponent, C chain**	**C1QC**	8.06	−5.65	−5.77
**CD74 molecule, major histocompatibility complex, class II invariant chain**	**CD74**	7.85	−5.6	−3.86
**Complement component 4A (Rodgers blood group)**	**C4A, C4B**	7.49	−5.76	−2.94
**Major histocompatibility complex, class I, B**	**HLA-B**	7.15	−5.21	−5.46
Lectin, galactoside-binding, soluble, 1 (galectin 1)	LGALS1	7.11	−4.4	−4.03
**Major histocompatibility complex, class II, DR beta 1, 3–5**	**HLA-DRB1, 3–5**	6.9	−4.72	−3.68
**Major histocompatibility complex, class I, A**	**HLA-A**	6.83	−5.56	−5.78
**Lymphocyte antigen 96 (MD-2)**	**LY96**	6.81	−5.1	−3.39
Retinoic acid receptor responder (tazarotene induced) 3	RARRES3	6.55	−5.23	−5.2
**Major histocompatibility complex, class I, F**	**HLA-F**	6.34	−4.65	−4.59
**Serpin peptidase inhibitor, clade G (C1 inhibitor), member 1**	**SERPING1**	6.01	−4.78	−3.79
**Chemokine (C-C motif) receptor 1**	**CCR1**	5.93	−4.47	−3.93
**Fc fragment of IgE, high affinity I, receptor for; gamma polypeptide**	**FCER1G**	5.71	−4.24	−3.4
**HLA-G histocompatibility antigen, class I, G**	**HLA-G**	5.67	−4.17	−4.15
**Major histocompatibility complex, class I, E**	**HLA-E**	4.91	−3.65	−4.06
Proteasome (prosome, macropain) subunit, beta type, 9	PSMB9	4.84	−4.32	−3.37
**Lectin, galactoside-binding, soluble, 3 binding protein**	**LGALS3BP**	4.27	−4.02	−4.32
Proteasome activator subunit 2 (PA28 beta)	PSME2	4.23	−4.39	−5.97
**Interferon-induced protein 44**	**IFI44**	4.19	−4.85	−4.25
**Interferon induced transmembrane protein 1 (9–27)**	**IFITM1**	4.15	−3.41	−4.29
**Serpin peptidase inhibitor, clade A (antitrypsin), member 3**	**SERPINA3**	4.09	−2.37	−3.57
**Tumor necrosis factor superfamily, member 13b**	**TNFSF13B**	4.07	−3.93	−4.46
**Interferon-stimulated transcription factor 3, gamma 48kDa**	**ISGF3G**	3.84	−4.25	−4.26
Dystrobrevin, alpha	DTNA	3.84	−3.3	−4.09
**Hepcidin antimicrobial peptide**	**HAMP**	3.74	−3.43	−3.96
**Secreted phosphoprotein 1 (osteopontin)**	**SPP1**	3.5	−3.18	−3.24
Vascular endothelial growth factor	VEGF	3.11	−3.01	−2.28
**Myxovirus resistance 1, interferon-inducible protein p78**	**MX1**	2.83	−3.6	−3.15
**Interferon-induced protein with tetratricopeptide repeats 3**	**IFIT3**	2.42	−4.93	−4.78
Homeodomain interacting protein kinase 2	HIPK2	−2.81	2.7	5.82
Multiple C2 domains, transmembrane 1	MCTP1	−4.54	6.38	3.24
RUN and FYVE domain containing 2	RUFY2	−9.07	9.76	8.37

† Redundant probes and unknown hypothetical proteins were removed. Genes are ranked using t-scores which were calculated by comparison with cognitively intact youngest-old (<86) subjects across of the 15 brain regions using Contrast analysis (GX™ Explorer, Gene Logic Inc.). Genes are arranged in order of decreasing t-scores in CDR0 subjects. Immune-response genes are in bold.

### Comparison of microarray data for two age categories using different normalization algorithms and different microarray platforms

The GeneChip U133AB platform data described above was normalized by MAS5. To further validate the microarray findings we re-analyzed the U133AB array raw dataset using a different normalization algorithm and performed microarrays of the same samples using the more advanced GeneChip U133 Plus2 platform. RMA normalization of the raw U133AB microarray data with subsequent CGEM based differential gene expression discovery using filtering condition similar to the MAS5 based analyses showed that although the results of the two analysis procedures differed with respect to the absolute number of differentially expressed genes identified (Table 7), the correlations between the two methods (r = 0.98, p = 0.003 for the numbers of differentially expressed genes) remained very high. Additionally, comparison of the MAS 5.0 normalization of U133AB array dataset with MAS 5.0 normalized values from the U133 Plus2 platform, also showed strong significant correlations between two datasets (r = 0.89, p = 0.04 for numbers of differentially expressed genes). Furthermore, pathway analysis indicated that affected genes were represented by the same set of pathways associated with immune responses irrespective of MAS5 vs. RMA analysis and U133AB or U133 Plus2 array platforms (Table 8). The top six affected pathways were common to the U133 Plus2 and the U133AB datasets. The rest of the pathways identified by the U133 Plus2 platform expanded the number of previously detected signaling pathways associated with immune function considerably.

**Table 7:** Number of significantly affected probe sets in U133AB and U133Plus2 microarray chips using MAS5 and RMA-CGEM –based analysis in two median age split cohorts.

Microarray Normalization/Platform	CDR0.5–1 86− vs CDR0 86−	CDR 4–5 86− vs CDR0 86−	CDR0 87+ vs CDR0 86−	CDR0.5–1 87+ vs CDR0 87+	CDR 4–5 87+ vs CDR0 87+
MAS5/*U133AB*	262	1071	332	132	1091
RMA-CGEM/*U133AB*	305	810	313	207	1023
MAS5/*U133 Plus2*	482	1875	979	382	1115

† Filtering conditions were as following: MAS5-based: Fold change ≥1.3; p-val (t-test) ≤0.05; presence >70%; RMA-CGEM: Fold change ≥1.3; *p*-val (ANOVA) ≤0.05; presence >70%.

**Table 8:** Comparison of the pathways affected in cognitively normal oldest-old compared to cognitively normal youngest-old analyzed on two different microarrays (U133AB and U133Plus2).

*Pathway Name*	*-Log(P-value) U133AB*	*Ratio U133AB*	*-Log(P-value) U133Plus2*	*Ratio U133Plus2*
**Antigen Presentation Pathway**	**6.76**	**4.36E-01**	**9.45**	**4.36E-01**
**IL-4 Signaling**	**3.05**	**1.47E-01**	**4.38**	**2.27E-01**
**Natural Killer Cell Signaling**	**3.05**	**1.00E-01**	**4.38**	**2.27E-01**
**Complement System**	**2.33**	**1.11E-01**	**2.75**	**2.78E-01**
**Acute Phase Response Signaling**	**2.21**	**7.56E-02**	**2.98**	**1.82E-01**
**Fc Epsilon RI Signaling**	**1.72**	**9.00E-02**	**2.95**	**2.08E-01**
Synaptic Long Term Potentiation	*ns*	*ns*	9.65	3.12E-01
Fcγ Receptor-mediated Phagocytosis in Macrophages	*ns*	*ns*	7.68	2.88E-01
Interferon Signaling	*ns*	*ns*	4.32	3.79E-01
GM-CSF Signaling	*ns*	*ns*	3.90	2.74E-01
Chemokine Signaling	*ns*	*ns*	3.89	2.53E-01
IL-8 Signaling	*ns*	*ns*	3.63	1.86E-01
IL-4 Signaling	*ns*	*ns*	3.51	2.43E-01
B Cell Receptor Signaling	*ns*	*ns*	3.35	1.92E-01

† Fischer's exact test was used to calculate p-values; the ratios are between the numbers of genes from the dataset that mapped to the pathway to the total numbers of genes in the canonical pathway. Common pathways between two microarray platforms are in bold font.

Acute phase response, which includes IL-6 signaling was among those affected in both microarray datasets in cognitively normal (CDR0) and severely cognitively impaired (CDR4–5) oldest-old subjects. Comparison of the genes involved in IL-6 signaling showed that more than half of the genes identified in U133Plus2 datasets were also detected as affected in U133AB datasets (Table 9). As evident from the high correlations described above, the directionality and magnitude of changes for those genes were very similar in both microarray platforms and comparison groups. Higher representation of differentially expressed genes in U133Plus2 dataset vs. those detected by U133AB can be explained, at least in part, by the fact that the Plus2 chip contains 10,000 more probe sets than the older U133AB chip as well as an improved detection sensitivity limit over the older version of the chip.

**Table 9:** Expression analysis of the immune response genes in the inferior temporal cortex - BA20.

Gene Symbol	CDR0 87+ vs CDR0 86− FC Ratio (*p*-value)	MD 87+ vs CDR0 87+ FC Ratio (*p*-value)	SD 87+ vs CDR0 87+ FC Ratio (*p*-value)	MD 86− vs CDR0 86− FC Ratio (*p*-value)	SD 86− vs CDR0 86−FC Ratio (*p*-value)
CD74	**2.28 (0.001)**	**−1.77 (0.002)**	−1.17 (ns)	**1.59 (0.027)**	**1.72 (0.031)**
LY96/MD-2	**2.26 (4-E4)**	**−1.76 (9-E5)**	**−1.24 (0.04)**	1.45 (ns)	**1.72 (0.048)**
HLA-DPA1	**1.65 (2-E4)**	**−1.53 (1-E4)**	**−1.26 (0.015)**	1.09 (ns)	1.10 (ns)
C1QC	**1.45 (0.027)**	**−1.52 (0.012)**	−1.20 (ns)	1.15 (ns)	1.22 (ns)
C3	**1.38 (0.013)**	**−1.76 (7-E5)**	−1.13 (ns)	−1.21 (ns)	−1.12 (ns)
TYROBP/DAP12	1.25 (ns)	**−1.48 (0.002)**	−1.14 (ns)	1.02 (ns)	1.07 (ns)
ITGB2	−1.20 (ns)	**−2.07 (1-E4)**	**−1.88 (0.002)**	−1.84 (ns)	−1.83 (ns)
CX3CR1	1.16 (ns)	**−1.74 (0.001)**	**−1.41 (0.049)**	−1.05 (ns)	−1.38 (ns)

Tissue samples from BA20 were analyzed by qPCR. FC Ratio-fold change ratios represent the ratio of means for each gene normalized to the geometric means of four housekeeping genes between controls and cognitively impaired patients. Significantly changed genes (p<0.05) are highlighted in bold. *ns*- non-significant.

### Quantitative PCR validation of microarray gene expression findings

**Figure 3:**
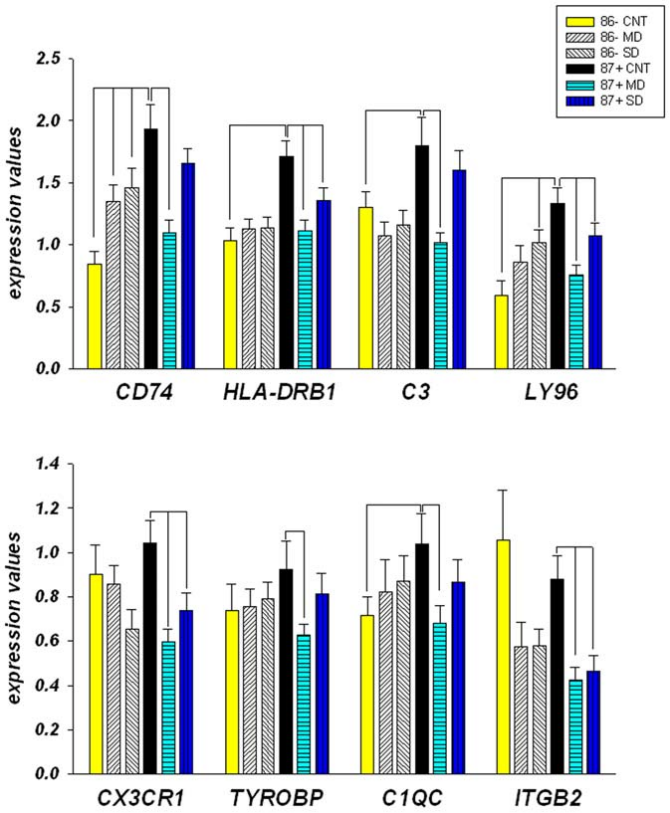
Relative mRNA expression of the immune system related genes in the inferior temporal cortex (BA20) of persons with dementia and cognitively normal (CDR0) individuals measured by qPCR. Subject characteristics are shown in Table 1. Group definitions are as indicated on the figure (MD  =  CDRs 0.5–1; SD  =  CDRs 4–5). Data is expressed as geometric means ± SEM of individual expression values normalized to the four housekeeping genes: B2M, GUSB, PPIA and RPLP0 as described in Methods. Significance is indicated by the comparison lines. Fold change values and *p-*values are shown in Table 9.

Microarray findings indicated that abnormal expression of antigen presentation, complement and inflammatory response genes was most closely associated with dementia and its progression in the oldest-old. We performed independent qPCR analysis of a small subset of 8 genes that includes CD74, HLA-DPA1, C3, LY96/MD-2, CX3CR1, TYROBP/DAP12, C1QC and ITGB2 in a study of 4 groups (Table 1; young-old CDR 0; young-old MD, oldest-old CDR 0; oldest-old MD) in the inferior temporal gyrus (BA20). These immune-related genes were selected for replication because they met high stringency filtering conditions (fold change ≥1.7; *p*(t-test)<0.001) in both MAS5 and RMA normalization algorithms in cognitively normal oldest-old individuals relative to the cognitively intact young-old donors in the microarray analysis. The results of this study are shown in Figure 3 and support the microarray findings by implicating the significantly increased expression of five genes: CD74, HLA-DPA1, C3, C1QC and LY96 (Table 9. All *p*s≤0.013) in the temporal cortex of cognitively normal oldest-old individuals relative to the cognitively intact young-old donors. It is important to note that the microarray analyses were based on global changes across all brain regions examined, it is therefore not surprising that only 5 of 8 transcripts evidenced differential expression in the one brain region studied by qPCR. Furthermore, the expression of all eight tested genes was significantly downregulated in cognitively impaired oldest-old individuals with MD (Table 9) relative to age matched cognitively intact donors. The expression of these genes was persistently downregulated in oldest-old individuals (Table 9) relative to age-matched cognitively intact donors, although only four: HLA-DPA1, LY96, CX3CR1 and ITGB2 reached conventional level of significance (p≤0.05). In contrast, in young-old individuals with dementia only two of these immune-associated genes were statistically significantly changed, CD74 and LY96, and consistent with the microarray findings and in contrast to oldest-old persons, their expression was upregulated relative to cognitively intact young-old individuals.

These results validate the microarray findings which showed increased expression of immune/inflammatory genes in the brains of cognitively intact oldest-old persons relative to cognitively intact young-old persons and decreases in cognitively compromised oldest-old persons relative to cognitively intact oldest-old controls.

### Expression of key inflammatory/immune system associated proteins in young-old and oldest-old persons with and without dementia

**Figure 4:**
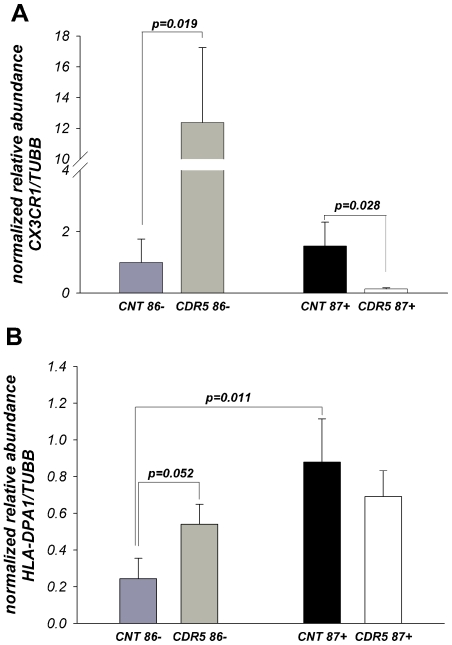
Relative abundance of CX3CR1 (A) and HLA-DPA1 (B) proteins in the inferior temporal cortex –BA20 of patients with SD (CDR4–5) and cognitively normal (CDR0) individuals. Protein levels of CX3CR1 and HLA-DPA1 were measured by Western Blot (Suppl. Fig. 2 and 3). Data expressed as means ± SEM of ODs normalized to the generic sample – “calibrator” and endogenous control - TUBB protein (N = 9–10/group, see Table 1).

Three of the most affected immune genes in the oldest-old: CX3CR1, HLA-DPA1 and CD74; were selected to assess whether their protein levels were similarly affected as their gene expression in the inferior temporal cortex. Figure 4 shows the results of quantitative Western blot analyses (Fig. S2 and Fig. S3) of the microglia-associated chemokine-fractalkine receptor CX3CR1 [42] and HLA-DPA1 (major histocompatibility complex, class II, DP alpha1). CX3CR1 protein levels were significantly increased in young-old persons with SD (CDR4–5, p = 0.019), but significantly decreased (p = 0.028) in the oldest-old SD persons. Although in this limited sample the increase in the level of CX3CR1 protein in cognitively intact oldest-old persons did not reach statistical significance, its levels were nominally elevated in the inferior temporal cortex. HLA-DPA1 protein levels were significantly increased in young-old persons with SD (p = 0.052) and in cognitively intact oldest-old individuals (p = 0.011), and showed a tendency to decrease in the oldest-old SD patients. Thus, the changes in protein expression corroborate the same pattern of gene expression found in the microarray and qPCR studies.

**Figure 5:**
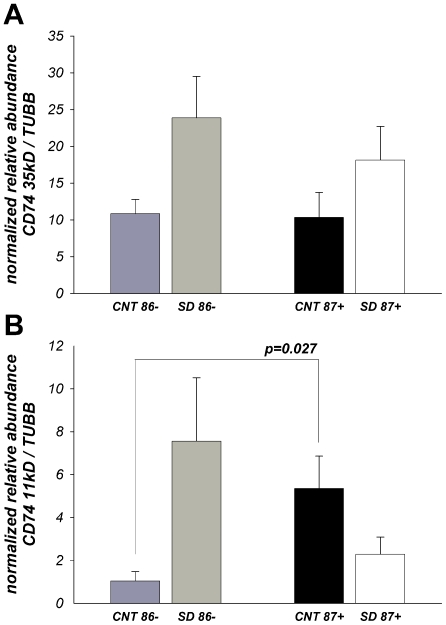
Relative abundance of CD74 -35/33kD (A) and -11kD (B) proteins in the inferior temporal cortex –BA20 of patients with SD (CDR4–5) and cognitively normal (CDR0) individuals. Protein levels of CD74 were measured by Western Blot (Suppl. Fig. 4). Data expressed as means ± SEM of ODs normalized to the generic sample – “calibrator” and endogenous control - TUBB protein (N = 9–10/group, see Table 1).

Figure 5 shows the results of quantitative Western blot analyses of the invariant gamma chain of MHC class II protein CD74 antigen (Fig. S4), which acts both as a chaperone for MHC class II molecules and as a receptor for the macrophage migration inhibitory factor (MIF). Western blotting with CD74 monoclonal antibody recognizing the cytoplasmic tail detected multiple bands ranging from 11 to 72kD with the main bands corresponding to MWs: ∼35/33, 41 and 11 kD. CD74- 33/35kD (Fig. 5A) as well as total CD74 (data not shown) showed a tendency to increase in patients with severe dementia in both age categories. In contrast to 35/33kD CD74, a small molecular size band (∼11kD), corresponding to the intracellular cleaved cytoplasmic domain of CD74 [43] that can function as an activator of NF-kB transcription factor and B-cells enriched co-activator-TAF(II)105, showed the same pattern of changes as CX3CR1 and HLA-DPA1 (Fig. 5B).

These results supported the observation that the expression of genes and proteins associated with the inflammatory/immune system is i) significantly increased in cognitively intact oldest-old persons relative to cognitively intact young-old persons; and ii) impaired in cognitively compromised oldest-old persons relative to cognitively intact oldest-old controls.

## Discussion

The notion that AD with its hallmark neuritic plaque, neurofibrillary tangle, and neurochemical system lesions is the preeminent cause of dementia in the elderly as been among the cornerstones of our conceptualizations of aging and dementia. This understanding has been supported by the nearly ubiquitous presence of AD pathological changes in age-related dementia as well as the near exponential increase of AD prevalence after 65 years of age. Increasingly, neuropathological and neuroanatomical studies (see introduction) have called this long-held tenet into question by suggesting that the neuropathological and neurobiological substrates of dementia can vary considerably between younger old vs. oldest-old persons. These latter observations have become increasingly salient with the knowledge that the oldest-old represent the fastest growing segment of the aging population with their numbers expected to reach above 19 million by the year 2050. The current study sought to identify some of the molecular biological substrates of dementia in the oldest-old and to determine how the neurobiology of dementia may change with increasing age. We examined gene expression changes across multiple brain regions in oldest-old individuals with or without cognitive impairment and compare them to those in more conventionally studied young-old persons. Our findings suggest that immune/inflammation-associated systems of the brain occupy a central role not only in the processes related to cognitive impairment in the oldest-old, but also with the preservation of cognitive function and healthy aging in extreme old-age. The results reported here also support the emerging neuropathology literature in the oldest-old population by suggesting that often the neurobiological substrates of dementia in the oldest-old may be qualitatively different than those prevalent in younger old persons.

Analysis of a large microarray dataset identified a lack of significant overlap in differentially expressed gene sets associated with dementia in young-old vs. oldest-old persons. The top of the list of significantly altered signaling pathways in young-old persons with dementia was well represented with pathways associated with neurotransmission and synaptic function: GABA and glutamate receptor signaling, synaptic LTP and neural development, including genes participating in neuregulin, IGF1, VEGF signaling, axonal guidance and calcium signaling. As expected from a plethora of published studies, the majority of genes from these pathways were downregulated in cognitively impaired individuals. Other affected pathways included networks of genes previously implicated in brain aging: IGF1, glucocorticoid receptor, nitric oxide and NF-kB signaling, cell cycle regulation, oxidative stress response, and pathways associated with neurodegeneration: hypoxia, Huntington's and Parkinson's diseases and amyotrophic lateral sclerosis signaling. These data are consistent with previous genome-wide gene expression studies of different brain regions from persons with dementia and AD with designs that did not distinguish between young-old and oldest-old cases and utilized brain specimens from persons the majority of whom fall within the young-old age range of the current study [37]–[40], [44].

In contrast to young-old persons, oldest-old subjects with mild dementia evidenced predominant abnormalities in pathways associated with the immune response system including antigen presentation, classic complement system, and oxidative phosphorylation. Also in contrast to young-old subjects, oldest-old subjects with dementia show under representation of pathways and gene ontology (GO) categories related to neuronal function and were limited only to LTP. These results are in agreement with data derived from neuropathology studies indicating that oldest-old individuals with AD display striking resistance to at least some neurodegenerative processes and only mild neuronal loss in the hippocampus [13], underscoring the limits of neuropathological “lesion” hypotheses of AD and dementia progression in this particular age group [28], [45].

We performed analyses of the microarray data sets to address the question of the expression of genes that are important to both healthy aging, i.e., as people age from young-old to oldest-old without dementia, and to dementia irrespective of age. The expression of 65–75 transcripts was changed in all 5 groups (cognitively intact oldest-old; cognitively compromised young-old (mild and severe dementia) and cognitively compromised oldest-old (mild and severe dementia)) relative to the cognitively intact young-old index group (Fig. 2, Table 6). The vast majority of these genes were related to immune function. Interestingly, while the expression of some of these genes was increased in young-old persons with dementia, consistent with the previous reports on AD [46]–[52], the expression of the majority of immune response genes was down-regulated in the studied brain regions of cognitively impaired oldest-old persons. Furthermore, the expression of these immune-system related genes was increased in the brains of cognitively intact oldest-old persons. Interestingly, the upregulation of immune and inflammatory system genes that we have noted in the oldest-old are similar to those of a recent microarray study of gene expression across the lifespan in four brain regions [35].

Nine genes, members of major histocompatibility complex (MHC) classes I and II, were the most prevalent group among differentially expressed genes in cognitively normal oldest-old individuals. These molecules are central to antigen presentation processes to T-cells suggesting the presence of antigen presenting cells capable of initiating primary or secondary immune responses. In the CNS, MHC class II immunoreactivity is abundantly detected in activated microglia, in lymphocytes within the neuropil and scarcely in astrocytes in sporadic AD, and is also constitutively expressed by microglia in the white matter during normal aging [53], [54]. Increased expression of Toll-like receptor, TLR4 and lysosomal associated membrane protein - LAMP2 in cognitively normal oldest-old individuals detected in the microarray analyses (data not shown) corroborate expression of MHC genes and suggests activation of the initial steps in antigen processing and presentation, such as acquisition of antigens and recruitment of phagolysosomes necessary for proteolysis of antigenic peptides. Whether upregulation of MHC class I and II genes in cognitively normal oldest-old individuals is functionally associated with antigen presentation or is involved in interactions between neural and glial cells needs further investigation.

The CX3CR1, fractalkine receptor is expressed by a wide spectrum of hemopoietic cells in the periphery. In the CNS, CX3CR1 was first described on microglia [55] and later in neurons [56]. Since fractalkine, the exclusive ligand for CX3CR1 is predominantly expressed by neurons the factalkine-CX3CR1 complex may be central for neuron-microglia communication [57]. It has been suggested that the expression of CX3CR1 has beneficial neuroprotective and neuronal survival consequences in animal models of systemic inflammation, Parkinson disease and amyotrophic lateral sclerosis [58]. These beneficial effects may result from fractalkine-CX3CR1 elicited signaling leading to the suppression of pro-inflammatory cytokines and ROS production [59], [60].

DAP12/TYROBP and LY96 were among the genes whose expression was significantly affected in successful aging and in dementia in oldest-old persons, albeit in different directions. DAP12/TYROBP is associated with a large family of receptors including those that recognize MHC class I molecules in hematopoietic cells: dendritic cells, monocytes, macrophages, natural killer cells, and some B and T cells. The importance of DAP12 in brain function is demonstrated by the fact that loss-of-function mutation of DAP12 is associated with a rare presenile dementia known as Nasu-Hakola disease [61]. LY96/MD-2 is a component of a tri-molecular complex consisting of TLR4, LY96 and CD14 which is a critical effector of microglial activation. The potential importance of this complex to AD-associated dementia is suggested by in vitro experiments that have shown that impairment of this complex inhibits microglial and monocytic activation by aggregated Aβ peptide [62].

Upregulation of two components of the classical complement system - C3 and C1Q suggests initiation of the complement cascade which ultimately leads to formation of the membrane attack complex (C5b-9) that targets affected cells for lysis [63]. Activation of the classic and alternative complement pathways during progression of AD has been detected in numerous studies (for review see [64]) and may mediate, at least in part, inflammation and neurodegeneration in AD. The complement and innate immune system, however, may have dual, beneficial and harmful, effect on CNS [47], [64]. Harmful effect, when it causes extensive tissue damage, or protective, when it binds and removes toxic cellular debris, protein aggregates and/or pathogens. To protect cells against potentially harmful action of activated complement complexes cells are equipped with complement inhibitors, such as SERPING1, a C1 inhibitor, which belongs to a large family of serine protease inhibitors (serpins) and inhibits the proteolytic activity of C1 subcomponents C1r and C1s [65]. Consistent with its protective function, gene expression of SERPING1 was increased in cognitively normal oldest-old subjects and downregulated in demented subjects from the same age category (Table 5).

The source of the immune related gene expression changes in oldest-old persons is not directly discernable from the current data set. The lack of detectable signals for T-cell receptors, which are central for antigen presentation to T cells in our microarrays, refutes the presence of infiltrating CNS peripheral T cells. Immune related gene expression changes could be attributable to expression changes in microglia, astrocytes, neurons or vascular elements. Only future detailed immunohistochemical studies can address this question fully. However, we note that there were no differences between levels of conventionally accepted markers for astrocytes (GFAP- glial fibrillary acidic protein, PEA15-phosphoprotein enriched in astrocytes,15kD, ApoE and aquaporin 4), oligodendrocytes (MBP, MAG, CNP, SOX10, OLIG2 and PLP), neurons (ENO2, NEFL and NEFH) or microglial (CD68, CD11 and MMP12) markers between young-old and oldest-old individuals.

The abundant expression level of four identified immune-related genes (HLA-DPA1, CD74, CX3CR1 and C3), their significant correlations with L-ferritin (*r*≥0.7; *p*<0.01), a gene predominantly expressed in microglia [66] and lack of significant positive correlations with neuronal, oligodendrocytes and astrocytes markers, suggests an association of both cognitive health and compromise with CNS resident microglial cells, rather than with infiltrating peripheral hemopoietic cells. In response to injury, quiescent microglial cells with characteristics of immature myeloid lineage cells, undergo rapid morphological and functional transformation and acquire properties of mature myeloid cells, including antigen presentation, matrix metalloproteinase production and phagocytosis, as well as cytokine and growth factor secretion. These properties of microglia may confer neuroprotective or neurotoxic effect to neural tissue. Several identified genes in cognitively intact oldest-old subjects are known to encode proteins capable of protecting against microglial neurotoxicity in animal models. CX3CR1 null mice show more neuronal loss and cell-autonomous microglial neurotoxicity than control mice[58] and DAP12/TYROBP [67] or associated receptor TREM2 [68] null mice show impaired phagocytic activity and enhanced inflammatory gene expression that is manifested in adult onset dementing leukoencephalopathy caused by loss of function mutations of DAP12 or TREM2. Overall these observations suggest that cognitively intact oldest-old subjects may have developed microglial-mediated protection of neural cells against age–related microglial neurotoxicity and/or accumulated neurotoxic molecules, such as Aβ.

These microarray, qPCR and protein expression results strongly suggest that 1) the neurobiological substrates of dementia in young-old individuals are not identical to the neurobiological mechanisms that underlie dementia in oldest-old persons; 2) the predominant genes whose expression is affected in demented young-old persons comprise those associated with neurotransmission and synaptic function, whereas affected neurobiological pathways in oldest-old demented persons are more likely to be associated with immune/inflammatory responses of the CNS; and 3) the brains of cognitively intact persons who survive to advanced old age, the oldest-old, express a select set of immune/inflammatory genes at a higher level than young-old persons with intact cognition. These analyses also support the neuropathological study findings described above by suggesting that the neurobiological processes that subserve the development and progression of dementia in “conventional” young-old subjects have features that are fundamentally different from those associated with dementia in the oldest-old. One parsimonious interpretation of the expression of immune system marker genes in the oldest-old is that a robust immune system is essential for the maintenance of cognitive health to very old age [35], [69], [70], and that a failure of this system to respond to activating stimuli may contribute to the development of dementia in the oldest-old.

## Materials and Methods

### Ethics Statement

Diagnostic and postmortem consent procedures were approved by the institutional review boards of Mount Sinai Medical Center, Jewish Home and Hospital and J.J. Peters VA Medical Center. Consents for brain donation were obtained in writing from the legal next of kin of all donors.

### Sample Information and Preparation of Total RNA

Brain tissue specimens were derived from the Brain Bank of the Department of Psychiatry of the Mount Sinai School of Medicine (New York, NY)/J.J. Peters VA Medical Center (Bronx, NY). The precise tissue handling procedures have been described in detail.[14], [15], [31], [71] Cerebral cortical regions (approximately 0.8–1 cm^3^) limited to grey matter from the frontal cortex (Brodmann areas BA: 8 (superior frontal gyrus), 10 (frontal pole), 44 (inferior frontal gyrus), 46 (dorsolateral prefrontal cortex), 4 (precentral/gigantopyramidal), anterior cingulate (ACG, BA: 24/32 – at the level of the genu of the corpus callosum)), posterior cingulate cortex (BA: 23/31 – at the level of the pulvinar), parietal cortex (BA: 7 – superior parietal lobule), temporal cortex (BA: 20 (inferior temporal gyrus), 21 (middle temporal gyrus), 22 (superior temporal gyrus), 36/28 (parahippocampal gyrus/entorhinal cortex)), 38 (temporopolar) and occipital cortex (BA: 17 – primary visual cortex) regions and from the hippocampus (at the level of the red nucleus) were dissected from flash frozen coronal sections, pulverized at −80°C and aliquoted. Aliquots (50 mg) from each region were used for microarray gene expression analysis. RNA isolation and preparation for the microarrays were as described previously [72], [73]. Similarly prepared aliquots from the BA20 (inferior temporal gyrus) were used in qPCR [74] and Western blot analyses.

All subjects died of natural causes with no history of licit or illicit drug abuse or neurological disease. Cognitively intact subjects (CDR0) with no evidence of neurological or neuropsychiatric diseases were matched with dementia subjects by age, postmortem interval (PMI) and brain pH (Table 1). The predominant causes of death were cardiovascular disease and myocardial infarction, cancer, septicemia and bronchopneumonia. Brain specimens from subjects who were comatose for more than 6 hours prior to death were excluded from the current study.

### Subject selection, cognitive assessment, neuropathological assessment and group stratification

All subjects were evaluated in detail for cognitive status during the last 6 months of life and the neuropathological assessment procedures were as previously described [14], [15], [71]. Fifty-three subjects were included in this postmortem study as the principal cohort. The subjects were selected from a large group of study participants who came to autopsy and had been residents of the Jewish Home and Hospital (JHH) in Manhattan and the Bronx, New York, other area nursing homes and assisted living facilities and the community. The cohort of subjects included in the microarray analysis was part of a larger clinical and epidemiologic study of early AD that has been extensively described in previous publications [31], [75]. Exclusion criteria were: presence of neuropathological lesions not associated with AD (including, but not limited to, Pick's disease, Lewy body inclusions, Parkinson's disease, stroke, multi-infarct dementia, and severe cerebrovascular disease judged to be sufficient to affect cognitive function [76], [77]), coma >6 hours prior to death, seizures or fever (>39°C) during the 24 hours prior to death, unnatural cause of death; and comorbid psychiatric disease such as schizophrenia. The Clinical Dementia Rating scale (CDR) [41] was used as the primary measure of dementia severity. Subjects were rated by the CDR to have no cognitive deficits (CDR = 0), questionable dementia (CDR = 0.5) and mild dementia -MD (CDR = 1.0) were combined in a single group, and severe to terminal dementia (SD) (CDR = 4.0–5.0) were combined in a single group (Table 1). A multi-step consensus-dependent approach was applied to the assignment of CDR scores based on cognitive and functional status during the last 6 months of life as described previously [14], [15]. When available, longitudinal neuropsychological assessment results were also considered in deriving the final consensus CDR score.

### Microarray Procedure and Data Analysis

Microarray analysis was performed using Affymetrix (Santa Clara, CA) HG-U133AB set as well as HG-U133 Plus2 GeneChip® as described in the standard protocol outlined in the GeneChip® Expression Analysis Technical Manual (Affymetrix Inc., Santa Clara, CA) and reported previously [31], [72], [73]. Each human sample RNA was processed and run on separate U133AB chip sets and U133 Plus2 chips. Data were normalized using MAS 5.0 algorithms and analyzed using the GX™ Explorer v.3.0 (Gene Logic Inc., Gaithersburg, MD) tools (expression, comparative and contrast analysis) as described previously [72], [73]. Each individual microarray/sample-region (15 brain regions) was treated as an individual sample, though comparison analysis and t-score calculation were performed by brain region. Microarray analysis of multiple brain regions was an advantage in our study over single region comparison. As it has been reported previously by our groups and others [31], [78], the degree of gene expression change in different cortical regions fluctuates and depends to a significant degree on disease severity. Gene expression change was considered to be significantly altered if the change in expression met the following criteria of p<0.05 relative to the expression level in the control group; fold change ≥1.3, present calls ≥70%. To validate the results obtained by MAS 5.0 analysis the raw microarray data from 2147 HG-U133A and B individual chips were transformed and analyzed using GeneSpring GX 7.3.1 (Agilent Technologies/Silicon Genetics, Santa Clara, CA). Raw data were pre-normalized with Robust Multi-Chip Average (RMA) [79], [80] with subsequent log transformation, normalization to the 50^th^ percentile of all values per chip and median-centered per gene using GeneSpring normalization. Statistical comparisons were made using GeneSpring's Cross Gene Error Model (CGEM), based on the deviation from 1.0 algorithm. The following filtering criteria were used in these GeneSpring/RMA/CGEM analyses: expression level ≥1; single factor ANOVA with confidence (*p*≤0.05) and fold change (≥1.4).[81] Genes accepted for subsequent validation by qPCR were those that met the high stringency criteria (fold change ≥1.7; p<0.001) by both MAS 5.0- and RMA based normalization of microarray analyses. Confirmatory microarrays using the same samples were additionally performed using newer version human genome arrays - Affymetrix HG-U133 Plus 2. The same MAS5.0 normalization and analysis procedures were applied to these datasets.

### Ingenuity Pathway Analysis

Datasets containing identifiers for the genes expressed (presence ≥80%) in the samples from the comparison analyses and corresponding differential expression scores (fold change values, t-scores and p-values) obtained from comparison analysis (MAS5.0 normalization, GX™ Explorer v.3.0, Gene Logic Inc.) were uploaded into the Ingenuity Pathway Analysis application. Differentially regulated genes (*p*≤0.05) were overlaid onto a global molecular network in the Ingenuity Pathways Knowledge Base (Ingenuity® Systems, www.ingenuity.com) to identify biological functions and canonical pathways. Ingenuity pathway analysis algorithmically generates networks of genes based on their connectivity. Fischer's exact test was used to calculate a p-value determining the probability that each biological function assigned to that dataset, or the association between genes in the dataset and the canonical pathway, was not due to chance alone. Significant (ps<0.05) canonical pathways were additionally ranked according to the ratios between the numbers of genes from the dataset that mapped to the pathway to the total numbers of genes in the canonical pathway. The scores for the networks were calculated based on the number of network eligible genes and the size of the network to approximate how relevant any particular network was to the total list of eligible genes.

### RT-qPCR

The mRNA levels of immune-related genes, which met selection criteria of both MAS 5.0- and RMA-based microarray analysis were measured by qPCR in a larger independent cohort from BA20 (inferior temporal gyrus - Table 1) using TaqMan® MGB probes and primer sets (Applied Biosystems, Foster City, CA).

For relative quantification of mRNA expression, relative values of examined genes calculated using the standard curve method were further normalized to geometric means (GMs) of endogenous control-genes as described previously [82]. Four housekeeping genes (GUSB, B2M, PPIA and RPLP0) were used as endogenous controls for human postmortem studies in BA20 and tested for the expression stability using geNorm (http://medgen.ugent.be/~jvdesomp/genorm/).

### Quantitative Western Blotting

Protein abundance was measured in the inferior temporal cortex, BA20 (Table 1), from severe dementia (CDRs 4–5) and CNT subjects (N = 10/group) from two age categories previously selected for microarray analysis using Western blotting.

Tissue specimens (50 mg sister aliquots to those used for qPCR) were homogenized in Urea/Tris solution: 50 mM Tris/HCl pH 7.4; 6 M Urea; 2% CHAPS containing 1 mM PMSF and cocktails of proteinase/phosphatase inhibitors (Pierce Biotech Inc, Rockford, IL). Total protein concentration in the tissue homogenates was determined with a CBQCA Quantitation Kit (Molecular Probes Inc, Eugene, OR). Aliquot samples of 15 µg of total protein in triplicates were loaded onto pre-cast 4–12% Tris-glycine gel (Invitrogen, Carlsbad, CA) under reduced conditions. A “standard-calibrator” (a mix of small aliquots of tissue from all samples) was used as a calibrator between the gels and run on each gel in triplicate. Blots were incubated with antibodies: rabbit anti-human CX3C chemokine fractalkine receptor 1, CX3CR1 (1∶500 v/v) from LifeSpan Biosciences Inc. (Seattle, WA); mouse anti-human HLA-DPA1 (1∶100 v/v) from Abnova (Walnut, CA); mouse mAb against human CD74 antigen protein (1∶1000 v/v), which recognizes cytoplasmic tail of CD74 (Cedarlane/StressMarq, Burlington, NC) and mouse anti-human β tubulin (TUBB; 1∶1000 v/v) – from Santa Cruz Biotech. Inc. (Santa Cruz, CA) or rabbit anti-human TUBB (Novus Biologicals, Littleton, CO). Electrophoresis, blotting, immunostaining, and infra-red (IR) fluorescence detection (IRDye 680 or 800 Goat Anti-appropriate species IgG, Li-Cor Biosciences, Lincoln, NE) were performed under standard conditions. Multiplex western blots were scanned on Odyssey Infrared Imaging System (Li-Cor Biosciences, Lincoln, NE). The linearity of the dose responses for the antibodies used was established in preliminary experiments. Images were analyzed and quantitated with Odyssey software ver.3 (Li-Cor Biosciences, Lincoln, NE). To account for gel to gel variability, the relative expression value (REV) of analyzed proteins in each sample was calculated as a ratio between the averaged intensities of the band in the experimental sample and in the “standard-calibrator”. Finally, relative values for examined proteins were normalized to endogenous control - TUBB.

### Statistical Data Analysis

Multiple statistical procedures were employed for different aspects of the study. Max *t*-scores, Pearson correlation coefficients and corresponding p-values (ANOVA) [83], [84] for each individual transcript were calculated by the contrast analysis of MAS5.0 normalized microarray data using the GX™ Explorer v.3.0 (Gene Logic Inc., Gaithersburg, MD). T-scores were used as a standardized measure of gene expression change for each individual transcript across all of the analyzed brain regions and described in detail previously [72], [75]. Contrast analysis is an extension of the fold change algorithm which takes in account variability and estimates how well individual gene expression patterns fit a specified model (the contrast pattern vector). The contrast pattern vectors were set up in a way to outline the increase of expression levels in tested sample set. Accordingly, larger positive scores together with significant (*p*≤0.01) and Pearson correlation coefficients meant that the pattern of variation of expression values between sample sets closely follows the pattern represented by the contrast vectors, indicating upregulation of gene expression. Large negative *t*-score values together with significant (*p*≤0.01) negative Pearson correlation coefficients meant that the pattern of variation is the inverse to the pattern represented by the contrast vectors, indicating downregulation of gene expression. Finally, *t-*scores close to zero meant that the gene's expression pattern matches neither the contrast pattern nor its inverse, or that the amount of variation between sample sets is comparable to or smaller than the variation within sample sets.

Differences in expression between CNTs and different groups of demented persons in the GeneSpring/RMA/CGEM analyses were examined using a one-way analysis of variance (ANOVA) with Benjamini and Hochberg multiple testing/false discovery rate corrections.[81] A two-tailed Student's *t*-test was used to compare relative mRNA expression of analyzed genes in qPCR experiments and relative abundance of proteins in Western blots. Student's *t*-test and correlation analyses were performed using Statistica (release 6.0).

## Supporting Information

Figure S1 Heat map of differentially expressed 75 common probe sets in YO and OO cognitively intact (CDR0) groups and cognitively impaired OO groups. Depicted are 75 probe sets (Fig. 2) identified by comparison between cognitively intact individuals YO (86−) and OO (87+) cohort. CDR0 groups are controls and SD group includes individuals with severe dementia (CDR4–5). Individual intensities of differentially expressed probesets from two brain regions: BAs 20 and 32. were standardized to have a mean of 0 and standard deviation 1 by linear transformation. Transformed data were ordered by the Cluster software, v.3.0. Each row represents a single transcript. Column sets represent comparison groups as indicated at the top of each set. The color scale extends from −1.8 to +1.8. Red part of the scale indicates positive values. Blue part of the scale indicates negative values.
(0.19 MB PNG)

Figure S2 Western blots of CX3CR1 and beta tubulin -TUBB in inferior temporal cortex (BA20). Signal for CX3CR1 appeared as a doublet/triplet band ∼50kD (under reducing conditions). Extended boiling (up to 5 hours) of protein extract in the presence of 2-mercaptoethanol eliminate these bands, and CX3CR1 appears as a single band ∼50kD. Optical density was measured for all of the bands.
(0.45 MB TIF)

Figure S3 Western blots of HLA-DPA1 and beta tubulin -TUBB in inferior temporal cortex (BA20). Signal for HLA-DPA1 appeared as a doublet band ∼29kD (under reducing conditions). Extended boiling (up to 5 hours) of protein extract in the presence of 2-mercaptoethanol eliminate doublet, and HLA-DPA1 appears as a single band ∼29kD. Optical density was measured for all of the bands.
(0.14 MB TIF)

Figure S4 Western blots of CD74 and beta tubulin -TUBB in inferior temporal cortex (BA20). Signals for HLA-DPA1 appeared in multiple bands ranging from 11 to 72kD with the main bands corresponding to MWs: doublet 33/35, 41 and 11 kD (under reducing conditions).
(0.73 MB TIF)
