# Supplementary figures and images for: Gain in Brain Immunity in the Oldest-Old Differentiates Cognitively Normal from Demented Individuals
Source: PLoS One. 2009 Oct 29;4(10):e7642. doi: 10.1371/journal.pone.0007642 (PMC2764344; doi:10.1371/journal.pone.0007642)

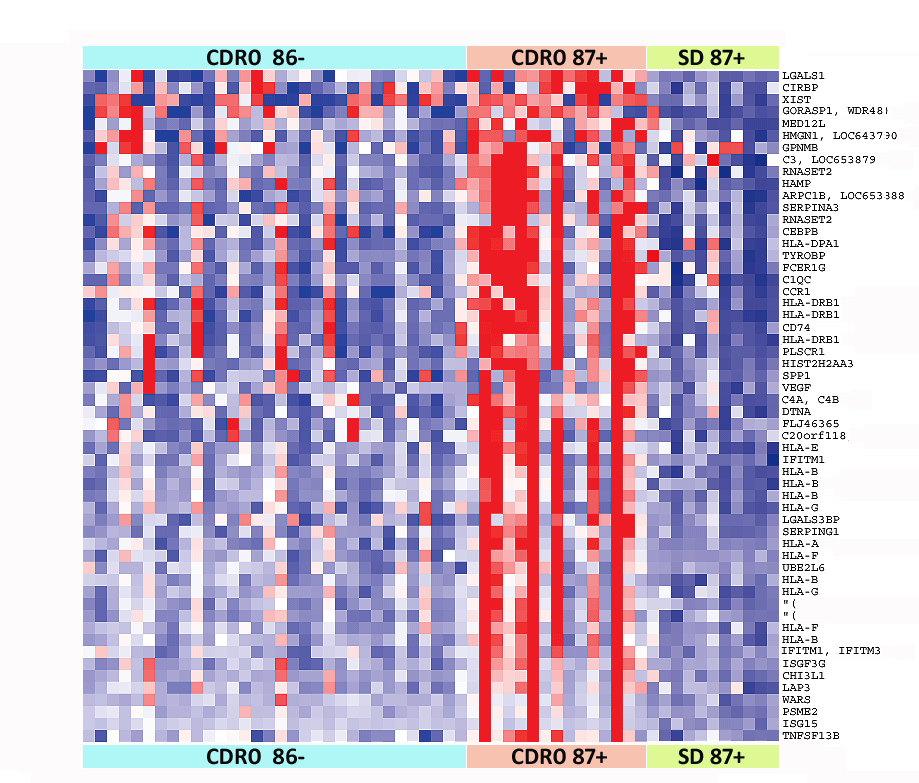

Supplement: Figure S1 — Heat map of differentially expressed 75 common probe sets in YO and OO cognitively intact (CDR0) groups and cognitively impaired OO groups. Depicted are 75 probe sets (Fig. 2) identified by comparison between cognitively intact individuals YO (86−) and OO (87+) cohort. CDR0 groups are controls and SD group includes individuals with severe dementia (CDR4–5). Individual intensities of differentially expressed probesets from two brain regions: BAs 20 and 32. were standardized to have a mean of 0 and standard deviation 1 by linear transformation. Transformed data were ordered by the Cluster software, v.3.0. Each row represents a single transcript. Column sets represent comparison groups as indicated at the top of each set. The color scale extends from −1.8 to +1.8. Red part of the scale indicates positive values. Blue part of the scale indicates negative values. (0.19 MB PNG) [file pone.0007642.s001.png]

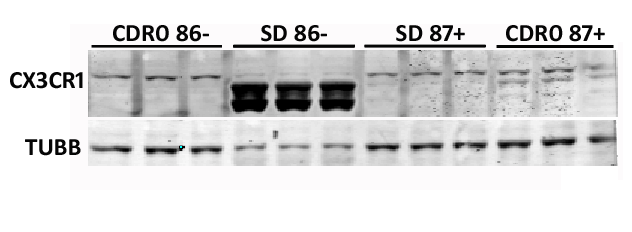

Supplement: Figure S2 — Western blots of CX3CR1 and beta tubulin -TUBB in inferior temporal cortex (BA20). Signal for CX3CR1 appeared as a doublet/triplet band ∼50kD (under reducing conditions). Extended boiling (up to 5 hours) of protein extract in the presence of 2-mercaptoethanol eliminate these bands, and CX3CR1 appears as a single band ∼50kD. Optical density was measured for all of the bands. (0.45 MB TIF) [file pone.0007642.s002.tif]

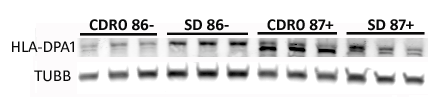

Supplement: Figure S3 — Western blots of HLA-DPA1 and beta tubulin -TUBB in inferior temporal cortex (BA20). Signal for HLA-DPA1 appeared as a doublet band ∼29kD (under reducing conditions). Extended boiling (up to 5 hours) of protein extract in the presence of 2-mercaptoethanol eliminate doublet, and HLA-DPA1 appears as a single band ∼29kD. Optical density was measured for all of the bands. (0.14 MB TIF) [file pone.0007642.s003.tif]

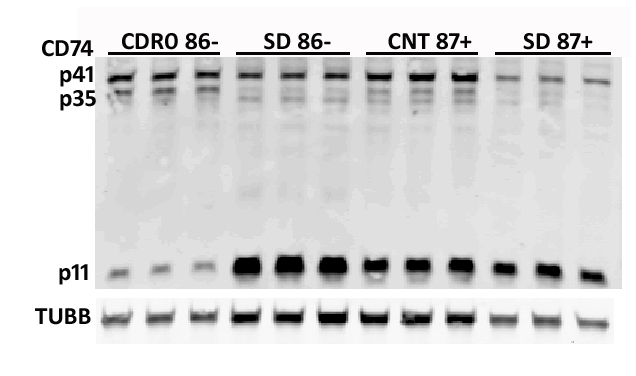

Supplement: Figure S4 — Western blots of CD74 and beta tubulin -TUBB in inferior temporal cortex (BA20). Signals for HLA-DPA1 appeared in multiple bands ranging from 11 to 72kD with the main bands corresponding to MWs: doublet 33/35, 41 and 11 kD (under reducing conditions). (0.73 MB TIF) [file pone.0007642.s004.tif]
